# Genome-wide association study for salinity tolerance at the flowering stage in a panel of rice accessions from Thailand

**DOI:** 10.1186/s12864-018-5317-2

**Published:** 2019-01-22

**Authors:** Chakkree Lekklar, Monnat Pongpanich, Duangjai Suriya-arunroj, Aumnart Chinpongpanich, Helen Tsai, Luca Comai, Supachitra Chadchawan, Teerapong Buaboocha

**Affiliations:** 1 Biological Sciences Program, Faculty of Science, Chulalongkorn University, Bangkok, Thailand; 2 Center of Excellent in Environment and Plant Physiology, Department of Botany, Faculty of Science, Chulalongkorn University, Bangkok, Thailand; 3 Department of Mathematics and Computer Science, Faculty of Science, Chulalongkorn University, Bangkok, Thailand; 4 Omics Sciences and Bioinformatics Center, Faculty of Science, Chulalongkorn University, Bangkok, Thailand; 5 Nakhon Ratchasima Rice Research Center, Rice Department, Ministry of Agriculture and Cooperatives, Nakhon Ratchasima, Thailand; 6 Department of Biochemistry, Faculty of Science, Chulalongkorn University, Bangkok, Thailand; 7 Department of Plant Biology and Genome Center, University of California Davis, Davis, CA USA

**Keywords:** Rice, Salt tolerance, Flowering stage, Genome-wide association study

## Abstract

**Background:**

Salt stress, a major plant environmental stress, is a critical constraint for rice productivity. Dissecting the genetic loci controlling salt tolerance in rice for improving productivity, especially at the flowering stage, remains challenging. Here, we conducted a genome-wide association study (GWAS) of salt tolerance based on exome sequencing of the Thai rice accessions.

**Results:**

Photosynthetic parameters and cell membrane stability under salt stress at the flowering stage; and yield-related traits of 104 Thai rice (*Oryza sativa* L.) accessions belonging to the *indica* subspecies were evaluated. The rice accessions were subjected to exome sequencing, resulting in 112,565 single nucleotide polymorphisms (SNPs) called with a minor allele frequency of at least 5%. LD decay analysis of the panel indicates that the average LD for SNPs at 20 kb distance from each other was 0.34 (*r*^2^), which decayed to its half value (~ 0.17) at around 80 kb. By GWAS performed using mixed linear model, two hundred loci containing 448 SNPs on exons were identified based on the salt susceptibility index of the net photosynthetic rate at day 6 after salt stress; and the number of panicles, filled grains and unfilled grains per plant. One hundred and forty six genes, which accounted for 73% of the identified loci, co-localized with the previously reported salt quantitative trait loci (QTLs). The top four regions that contained a high number of significant SNPs were found on chromosome 8, 12, 1 and 2. While many are novel, their annotation is consistent with potential involvement in plant salt tolerance and in related agronomic traits. These significant SNPs greatly help narrow down the region within these QTLs where the likely underlying candidate genes can be identified.

**Conclusions:**

Insight into the contribution of potential genes controlling salt tolerance from this GWAS provides further understanding of salt tolerance mechanisms of rice at the flowering stage, which can help improve yield productivity under salinity via gene cloning and genomic selection.

**Electronic supplementary material:**

The online version of this article (10.1186/s12864-018-5317-2) contains supplementary material, which is available to authorized users.

## Background

Stress caused by salinity is one of the most serious environmental factors, which inhibits plant growth and decreases crop productivity worldwide [1]. Primary effects occurring at the beginning of salt stress include retarded cell division and expansion [2], stomata closure and photosynthesis reduction [3]. During long-term exposure to salt stress, accumulation of salt ions in plant aerial parts via the transpiration stream leads to ionic stress [1, 2, 4]. To adaptively respond and survive under salinity, plants require changes of various cellular, physiological and metabolic mechanisms, which are controlled by the regulated expression of specific stress-related genes through cascades of complex regulatory networks [5–7].

Rice (*Oryza sativa* L.), one of the world’s most important cereal crops, is classified as a salinity sensitive crop [1, 8]. An electrical conductivity (EC) of ~ 6 dS m^− 1^ (~ 56.98 mM NaCl [9]) would result in more than 50% reduction in yield of many rice varieties [10]. Therefore, plant breeders are continuously improving salt tolerant rice cultivars to increase yield productivity [11]. However, salt tolerance is a multigenic trait, which underlying mechanisms are controlled by many genes and affected by the environment. Breeding efforts for developing salt tolerant rice have been limited because the salt tolerance mechanisms and the genes that control them are not completely understood [12–14]. To fill the knowledge gap between genotypes and phenotypes of the salt stress response in rice, forward and reverse genetics have been performed to identify salt-responsive loci/genes such as genetic mapping of quantitative trait loci (QTLs) using cross population; screening of mutants generated by chemical- or irradiation-induced mutagenesis; and transgenic approach [15, 16]. To identify salt-responsive genes using cross population, a number of mapping studies have identified QTLs of physiological traits related to salinity tolerance in economic crops such as soybean, barley and rice [17–19]. Although QTL mapping is a powerful and popular method to tag the salt tolerance region in plants, the examination of the variation is one of the limitation because QTL mapping can identify only allelic diversity that segregates between the parents of a particular F_2_ cross or within recombinant inbred lines and the mapping resolution is limited by the amount of the genetic recombination event occurring in the mapping populations [20, 21]. Moreover, the genotyping by SSR markers, which is usually based on polymerase chain reaction (PCR), is limiting to examining the kinds of variations, and laborious and time-consuming when high-density genotyping is needed for a large number of individuals [22].

Over the past several years, next generation sequencing has been used to rapidly generate a large amount of accurate genomic data, providing a powerful approach for functional genomics and molecular breeding studies, including the genome-wide association study (GWAS) [23]. GWAS, which is the analysis of the statistical association between genetic variants and traits on the whole genome scale in a large number of individuals within an organism, has been employed to identify causal genetic variability for target traits, including those in *Arabidopsis* and crop species [22, 24–26]*.* Compared with the QTL linkage mapping method, GWAS provides high resolution mapping using single nucleotide polymorphisms (SNPs) as genetic markers [22, 27]. GWAS in rice was performed for agronomic traits such as tiller number, grain width, grain length and spikelet number in the indica subspecies based on SNPs identified by whole-genome sequencing. [28, 29]. In another report, the genetic architecture of rice chlorophyll content at the heading stage was revealed by GWAS. Forty-six significant loci were identified and *Ghd7* was highlighted as a major locus for the natural variation of the chlorophyll content [30]. GWAS also revealed three QTLs (*qER1–3*) located on chromosomes 3, 6 and 12 associated with the responsiveness of yield-determination traits under field condition [31]. Application of GWAS for causative gene identification has been reported in rice responding to abiotic stresses such as aluminum, boron, cold, drought and salt stresses [32–36]. On salt stress, there are several GWA studies in rice with different growing stages and traits. Shi et al. [37] studies GWAS on germination stage of salt-treated rice using ~ 6000,000 SNPs, 11 loci containing 22 significant SNPs responsible for stress-susceptibility indices of the vigor index and germination time were identified. The strongest association region for germination time was detected on chromosome 1, near salt-tolerance QTL controlling Na^+^ uptake and K^+^ concentration. At tillering stage, GWAS was performed on rice exposed to short- (6 h), medium- (7 d) and long-term (30 d) salt stress based on ~ 200,000 SNPs. Around 1200 candidate genes associated with growth parameters, and Na^+^ and K^+^ content were identified [36]. For salt-treated rice at reproductive stage, only a study of Kumar et al. [38] were reported. Based on 6000 SNPs, it was shown that 20 loci were associated with the Na^+^/K^+^ ratio, and 44 loci were associated with other traits. Twelve association mappings with Na^+^/K^+^ were located on chromosome 1 where *Saltol*, a major QTL that controls shoot Na^+^/K^+^ homeostasis in rice at the seedling stage, is located. However, GWAS has not been applied for the analysis of photosynthetic and yield-related traits in rice exposed to salt stress at the flowering stage, which is a highly salt-sensitive stage. Additionally, no rice accession from Thailand where a large collection of diverse rice germplasms can provide new allelic diversity for salt tolerance [39], were analyzed by GWAS.

The objectives of this research were (1) to investigate and cluster Thai and Asian rice accessions based on physiological responses and yield-related traits under the salt-stress condition at the flowering stage and (2) to perform GWAS for these traits to identify regions/genes responsible for salt tolerance.

## Methods

### Plant materials and growing conditions

The association panel consisted of a diverse collection of 190 rice (*O. sativa*) cultivars including both standard salt-tolerant (Pokkali) and salt-sensitive (IR29) varieties. The rice accessions in this study were kindly provided by the Pathum Thani Rice Research Center (Additional file 1: Table S1). The experiment was designed with a randomized complete block design with four replications. According to the limitation of the time-consuming process of data collection, the experiment was performed in three separate sets of experiments. The standard salt-tolerant and salt-sensitive cultivars were included in every experimental set. Twenty-one day old seedlings were cultivated using a hydroponic system with WP No. 2 nutrient solution [40] and transplanted into pots containing soil (5 kg) and maintained until harvest. At heading stage in the flowering phase of each accession, water on the soil surface was drained before salt stress treatment. Rice plants were then watered with 900 mL of 150 mM NaCl solution to reach the desired final soil electrical conductivity (EC) of 8–9 dS m^− 1^ and treated for 9 days. For the control condition, rice plants were treated by tap water for the same period. Water level was kept at 2 cm above the soil surface throughout the experimental period. To recover, tap water was used to wash out salt ions in the soil every day until the soil EC was lower than 2 dS m^− 1^; this condition was maintained until harvest to collect yield-related traits. These experiments were conducted in the greenhouse facility at the Nakhon Ratchasima Rice Research Center, Rice Department, Ministry of Agriculture and Cooperatives. The air temperature was maximum at 32 °C with natural light and minimum at 21.1 °C during the night. The average relative humidity was 72.5%.

### Parameter collection for association analysis

Photosynthetic parameters consisting of net photosynthetic rate (*P*_N_), stomatal conductance (*g*_s_), transpiration rate (*E*), and intercellular CO_2_ concentration (*C*_i_) were measured during the same period (8:30–11:30 a.m.) by the LI-6400 XT portable photosynthesis system (LI-COR, Lincoln, NE) on the middle portion of the 2nd leaf (penultimate leaf) of the main tiller on days 0, 3, 6 and 9 after salt stress treatment. The photosynthetic photon flux density used was 1200 μmol photon m^− 2^ s^− 1^. The leaf temperatures and ambient CO_2_ concentration used during the measurement were 27–30 °C and 380 ppm, respectively. A method modified from Blum and Ebercon [41] was used for cell membrane stability (CMS) measurement. One gram of the 2nd leaf was cut into segments of 2 mm in length and put into 10 ml of deionized water in a test tube and left at room temperature for 2 h. Electrical conductivity (EC_1_) of the sample solutions was measured using the universal instrument for measurements of conductivity (SevenCompact™ conductivity S230, Metler, USA). Then, the tissues in the test tubes were boiled for 15 min, cooled to room temperature, and the final electrical conductivity (EC_2_, maximum conductivity of the tissues) was measured. The percent CMS was calculated as 100 × [100 – (EC_1_/EC_2_ ratio)]. For yield-related traits, the numbers of tillers (TIL), panicles (PAN), filled grains (FG) and unfilled grains (UFG) per pot were recorded at the end of experiment.

### Exome library preparation

Rice gDNA was extracted from leaf tissue using the Genomic DNA Mini Kit (Plant) (Geneaid Biotech Ltd., Taiwan) and the amount of DNA was quantified using a spectrophotometer. For the exome library preparation, gDNA was fragmented using dsDNA Fragmentase (New England Biolabs, Ipswich, MA). The sheared DNA was modified using an End Repaired enzyme (New England Biolabs) and deoxyadenosine was added at the 3′ end using a Klenow fragment (New England Bio-labs). Each of the unique DNA barcodes (Bioo Scientific, Austin, TX) was joined to DNA in each library using DNA ligase (New England Biolabs). Pre-capture libraries were hybridized with the capture probes of the rice exome region, which were designed based on the *O. sativa* ‘Nipponbare’ database (Michigan State University [MSU] Rice Genome Annotation Project). The capture libraries were cleaned using AMPure (Beckman Coulter, Indianapolis, IN) and amplified by PCR using post-capture primers. The final yields were quantified by Bioanalyzer (Agilent Technologies, Santa Clara, CA). Exome-capture libraries (18–23) were pooled in each lane and sequenced using the Illumina HiSeq2000’s protocol in the Illumina genome analyzer (San Diego, CA).

### Data analysis

#### Statistical analysis of phenotypic traits

Statistical analyses were performed with IBP SPSS ver.22 (IBM Corp., Armonk, USA). Analysis of variance (ANOVA) was carried out to assess the effects of genotype, environment, and G × E interactions using the general linear model procedure. Duncan’s Multiple Range Test was used to compare the mean value for tests of significance. Cluster analysis among physiological responses was performed by JMP ver. 11 (SAS Institute Inc., Cary NC, USA) and R ‘corrplot’ package [42].

#### SNP genotyping and genotype data analysis

The short-sequence reads from the Illumina Genome Analyzer were grouped into the correct categories using the pipeline created by Missirian et al. [43]. The rice reference genome was downloaded from the database (Ensemble version IRGSP-1.0), and indexed by SAMtools [44]. Raw reads were aligned against the reference genome using the Burrow-Wheeler Aligner (BWA version 0.5.7–1) [45]. Variants were called using genome analysis toolkit (GATK; version 3.3–0) [46]. Variants were filtered if they fitted the following criteria: to be called *heterozygous*, minimum coverage and minimum percentage of each of the two observed major basecalls were 5 and 20, respectively and minimum total coverage was 10; for a position to be called *homozygous*, minimum coverage was 6 or 3 if positions with the minimum coverage of 6 were present in at least 10 accessions. SNP density was visualized using R 'CMplot' (https://github.com/YinLiLin/R-CMplot).

#### Population structure and linkage disequilibrium analysis

To estimate the number of subgroups in the panel to select the appropriate statistical model for association between the phenotypic and genomic data, analysis of the population structure within the rice population was performed using EIGENSOFT version 6.0.1, which used principal component analysis (PCA) to model ancestry differences in a population [47, 48]. Population stratification was visualized by plotting the first two PCs.

Linkage disequilibrium (LD) analysis was assessed by computing the correlation (*r*^2^) in frequency across a pair of SNP loci. The *r*^2^ values between pairs of SNPs were calculated using the command in PLINK [49] --r2 -ld window-kb 2000 --ld-window 999999 --ld-window-r2 0. This command was used to calculate LD association among SNP pairs to a distance of 2000 kb. LD decay analysis were conducted by division of marker pairs within the 2000-kb region into bins of 20 kb and *r*^2^ values within each bin were averaged. To visualize the result, the *r*^2^ values were sorted and plotted against the physical distance [38].

### Association mapping

To identify loci underlying the genetic regulation of traits mentioned above, SNPs were removed from the analysis by PLINK 1.07 [49] if their minor allele frequency was less than 5% across the panel or the genotype was unknown for > 40% of the varieties. The resulting ungenotyped markers were imputed using Beagle 5.0 [50]. Genome-wide association (GWA) mapping was conducted using GEMMA software based on the SNP data and the phenotypic data [51, 52]. To visualize the association results, the quantile–quantile (Q-Q) plots of observed *p*-values were constructed against expected *p*-values and Manhattan plots were constructed with the chromosome position on the X-axis against –log (*p*-value) of all SNPs using the R ‘qqman’ package [53]. The *p*-value of SNP marker was corrected for multiple tests by calculating *q*-value (FDR adjusted *p*-value) of each trait. SNPs with the *q*-value lower than 0.05 was selected as significant marker.

### QTL analysis

The list of candidate genes from GWA mapping was compared with the salt QTL mapping that was previously reported by Hu et al. [54], Patishtan et al. [36] and summarized in TropGENE [55], Gramene and http://www.plantstress.com/files/qtls_for_resistance.htm#salinity.

## Results

### Phenotypic variation among Thai rice accessions under salt effect

**Fig. 1:**
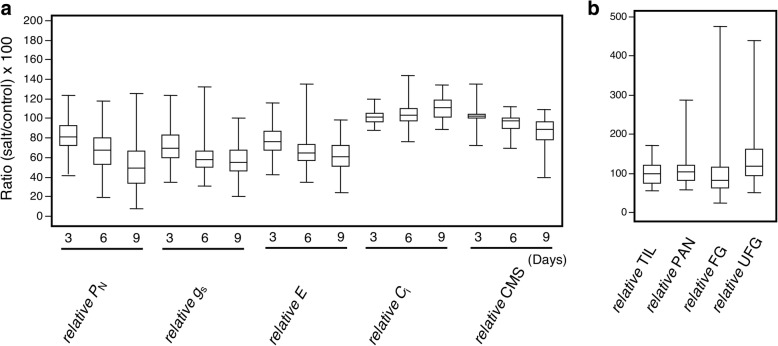
Box plots for relative phenotypic values (**a**) and yield-related traits (**b**) (calculated as percent phenotypic value in salt divided by control condition). The median of each trait is shown as a horizontal bar in the box, and the upper and lower sides of a box represent the first and third quartile values of the distribution, respectively. Whiskers represented maximum/minimum values. *P*_N_: net photosynthetic rate; *g*_s_: Stomatal conductance; *E*: Transpiration rate; *C*_i_: intercellular CO_2_ concentration; CMS: Cell membrane stability, TIL: number of tillers per plant; PAN: number of panicles per plant; FG: number of filled grains per plant and UFG: number of unfilled grains per plant

We evaluated photosynthetic parameters and cell membrane stability on 104 rice accessions individually at the flowering stage after salt stress for 3, 6 and 9 days and analyzed yield-related traits at harvesting time. The mean values and frequency distributions of all parameters of each accession are shown in Additional file 2: Table S2 and Additional file 3: Figure S1. The highest reduction of phenotypic traits was observed at day 9 after salt stress: photosynthetic rate, *P*_N_ (− 49%); stomatal conductance, *g*_s_ (− 50%); transpiration rate, *E* (− 43%), and cell membrane stability, CMS (− 18%) when compared with the control condition (Table 1). However, we found that the mean values of intercellular CO_2_ concentration, *C*_i_ increased about 6% at day 9 after salt stress treatment. For yield-related traits, on average, number of tillers per plant, TIL; number of panicles per plant, PAN; number of filled grains per plant, FG decreased by 19, 11 and 26%, respectively, whereas number of unfilled grains per plant, UFG increased by 10% (Table 1). To determine substantial genotypic variation in salt-stress responses, relative phenotypic values were calculated by the salt stability index of each rice accession [(salt/control) × 100] (Fig. 1). These parameters tended to decrease when plants were exposed to salt stress, except *C*_i_, which tended to increase under salt stress. The variations of phenotypic traits were found in all parameters and were pronounced, particularly in the case of *P*_N_, FG and UFG (Fig. 1).

**Table 1:** Mean and range of phenotypic values and yield-related traits of 104 rice accessions. Mean phenotypic values of control and salt-treated plants (*n* = 104) are shown with the S.D

Trait	DAT	Mean (Control)	Range	CV (%)	Mean (Salt treated)	Range	CV (%)
*P*_N_.day6 (μmol CO_2_ m^− 2^ s^− 1^)	3	11.50 ± 2.69	5.78–19.86	23.38	8.85 ± 2.78	3.28–16.40	31.42
	6	11.12 ± 3.07	5.20–19.89	27.61	7.06 ± 3.25	1.20–16.56	46.05
	9	10.35 ± 3.55	2.97–22.16	34.30	5.24 ± 3.49	0.17–14.36	66.62
*g*_s_ (mol CO_2_ m^−2^ s^−1^)	3	0.26 ± 0.09	0.11–0.56	36.73	0.17 ± 0.09	0.05–0.50	51.24
	6	0.22 ± 0.07	0.08–0.46	32.48	0.12 ± 0.04	0.04–0.22	33.88
	9	0.22 ± 0.09	0.08–0.48	40.23	0.11 ± 0.05	0.03–0.26	45.15
*E* (mmol H_2_O m^−2^ s^−1^)	3	3.57 ± 0.96	1.42–5.67	26.91	2.55 ± 0.69	1.16–4.21	26.85
	6	3.25 ± 1.14	1.19–5.62	35.18	1.98 ± 0.80	0.61–3.78	40.39
	9	3.11 ± 1.02	1.18–5.65	32.69	1.78 ± 0.72	0.66–3.58	40.25
*C*_i_ (μmol mol^−1^)	3	278.00 ± 30.33	214.95–335.38	10.91	268.11 ± 36.74	204.88–335.50	13.70
	6	271.96 ± 28.40	194.33–316.75	10.44	270.45 ± 31.82	205.16–327.75	11.77
	9	276.69 ± 24.59	218.85–315.75	8.89	292.42 ± 35.68	227.79–366.62	12.20
CMS (%)	3	88.33 ± 3.78	65.50–94.85	4.28	85.42 ± 5.70	55.99–93.33	6.67
	6	88.79 ± 3.48	76.06–95.26	3.92	80.13 ± 8.30	58.33–94.99	10.35
	9	86.63 ± 5.58	62.07–93.88	6.44	70.65 ± 14.07	29.54–90.83	19.91
TIL		4.97 ± 1.85	2.41–9.41	37.25	4.03 ± 1.02	2.58–7.77	25.31
PAN		3.14 ± 0.90	1.33–6.10	28.74	2.79 ± 0.63	1.67-5.17	22.68
FG		44.12 ± 28.68	1.31–121.50	65.01	32.78 ± 26.60	0.65-141.28	81.15
UFG		88.89 ± 33.55	26.67–186.56	37.74	97.82 ± 38.45	23.67–248.25	39.30

**Fig. 2:**
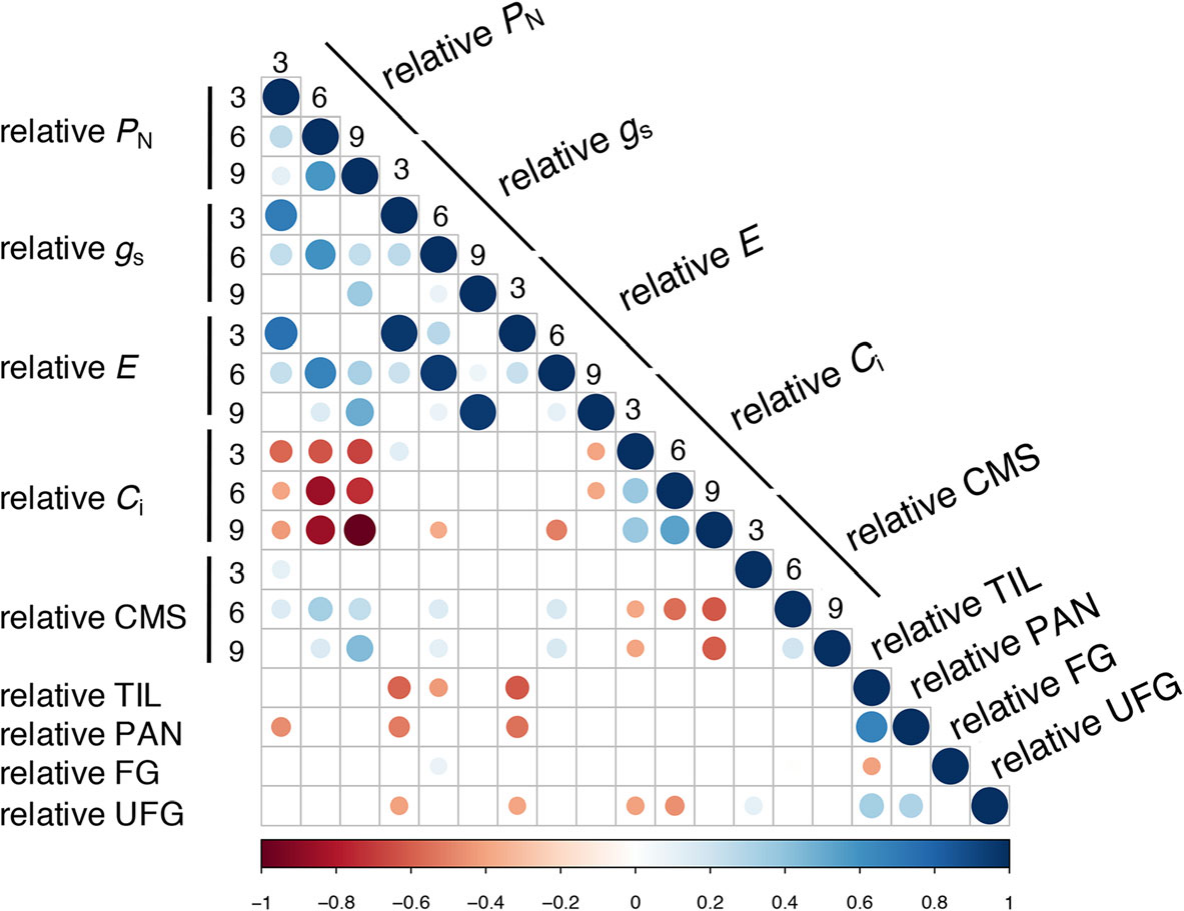
Pearson correlation coefficients computed for susceptibility indices of all traits and timings. *P*_N_: net photosynthetic rate; *g*_s_: stomatal conductance; *E*: transpiration rate; *C*_i_: intercellular CO_2_ concentration; CMS: cell membrane stability, TIL: numbers of tillers per plant; PAN: number of panicles per plant; FG: number of filled grains per plant and UFG: number of unfilled grains per plant. Cells with correlation values not significant at *p*-value < 0.05 are left blank

The relationships of the salt stability index of all parameters were determined by Pearson’s correlation *r* (Additional file 4: Table S3). We found a strong positive correlation between *P*_N_ and *g*_s_, or *E* (Fig. 2). *P*_N_ also had a positive correlation with CMS, though weaker, at days 6 and 9 after salt treatment. Conversely, a strong negative correlation between *P*_N_ and *C*_i_ was found. As expected for yield-related traits, the strongest positive correlation was observed between TIL, PAN and UFG. In addition, the relationship between photosynthetic performance and yield-related traits were observed. TIL was negatively correlated with *g*_s_ at days 3 and 6; and with *E* at day 3. Similarly, PAN was negatively correlated with *g*_s_ at day 3 as well as *P*_N_. Following the same trend, UFG was negatively correlated with *g*_s_ or *E* at days 3 after salt treatment, and with *C*_i_ both at days 3 and 6. (Fig. 2 and Additional file 4: Table S3). In an opposite trend, a positive correlation was found between FG and *g*_s_ at day 6. At day 9, no correlation was observed between photosynthetic parameters and yield-related traits.

### SNP data, population structure and LD pattern in the panel

**Fig. 3:**
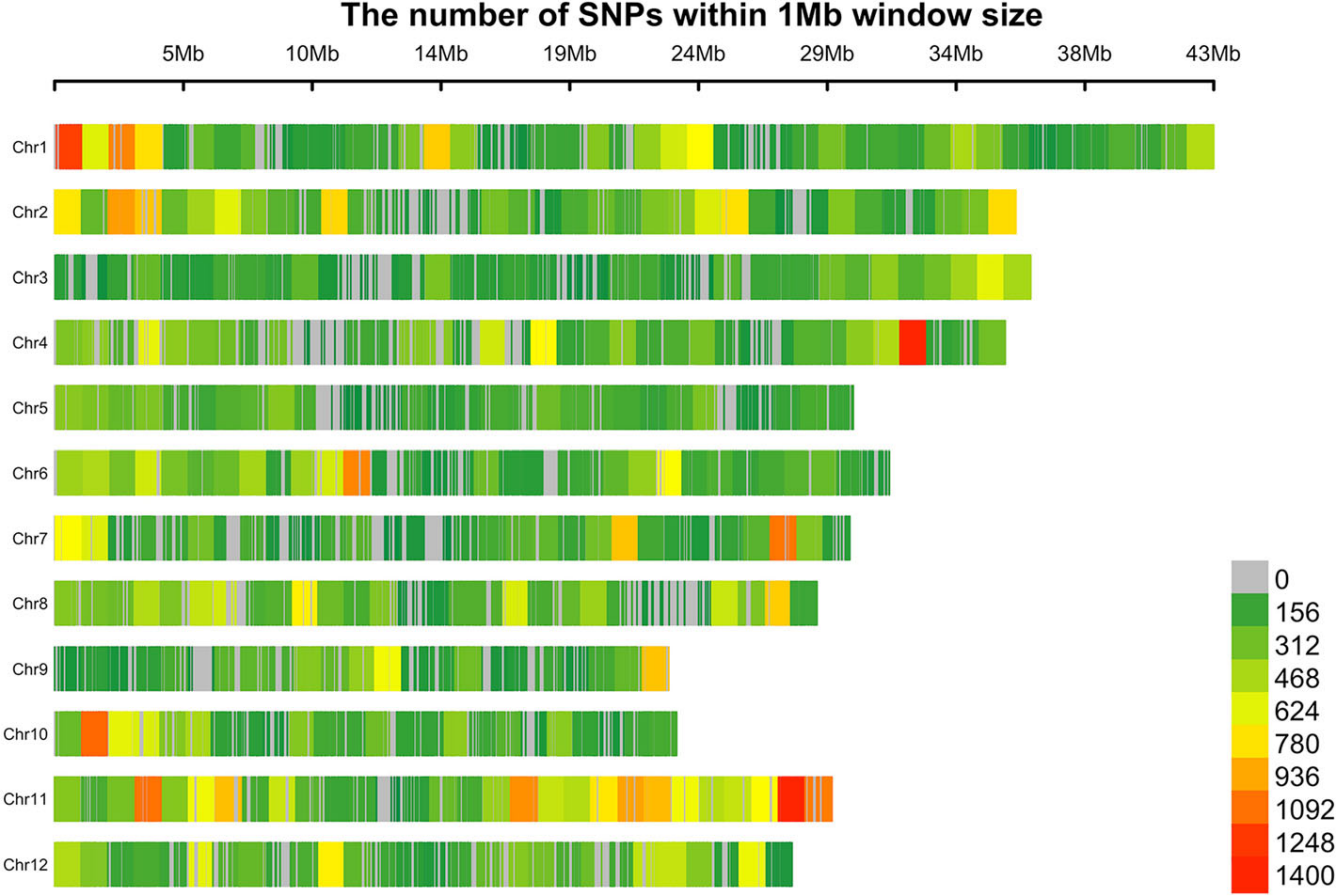
The number of SNPs called using GATK within 1 Mb window size in 12 rice chromosomes

The list of rice accessions used for exome sequencing is shown in Additional file 1: Table S1. In total, 190 rice accessions were used for exome-sequencing, with the capture probes designed to cover about 50 Mb of the nucleotide target covering all 12 chromosomes of rice. SNPs that showed a minor allele frequency (MAF) of < 5% of our population were removed to decrease overestimation of the effect of low-MAF SNPs. Therefore, the resultant number of 112,565 SNPs (Fig. 3), which were high-quality SNPs genotyped across this population, was subsequently used for GWAS.

EINGENSOFT was implemented for population structure analysis, which was based on PCA. Using SNPs identified by exome sequencing, two main subpopulations were delineated (Additional file 5: Figure S2), consisting of five accessions in the first group and 185 accessions in the second group, respectively. The rice accessions in the first group included ‘Ai Tai’, ‘Jao Haw’, ‘Beu Saw Mi’, ‘E-Puang’ and ‘Leung Tah Young’ rice, which were grouped as upland rice (Additional file 1: Table S1). We also found that ‘Pokkali’ rice, which is a standard salt tolerance variety, was separated from the two main sub populations. Therefore, before association analysis, we removed upland rice accessions to reduce strong subpopulation structure that may generate spurious association between the phenotype and unlinked SNP markers.

**Fig. 4:**
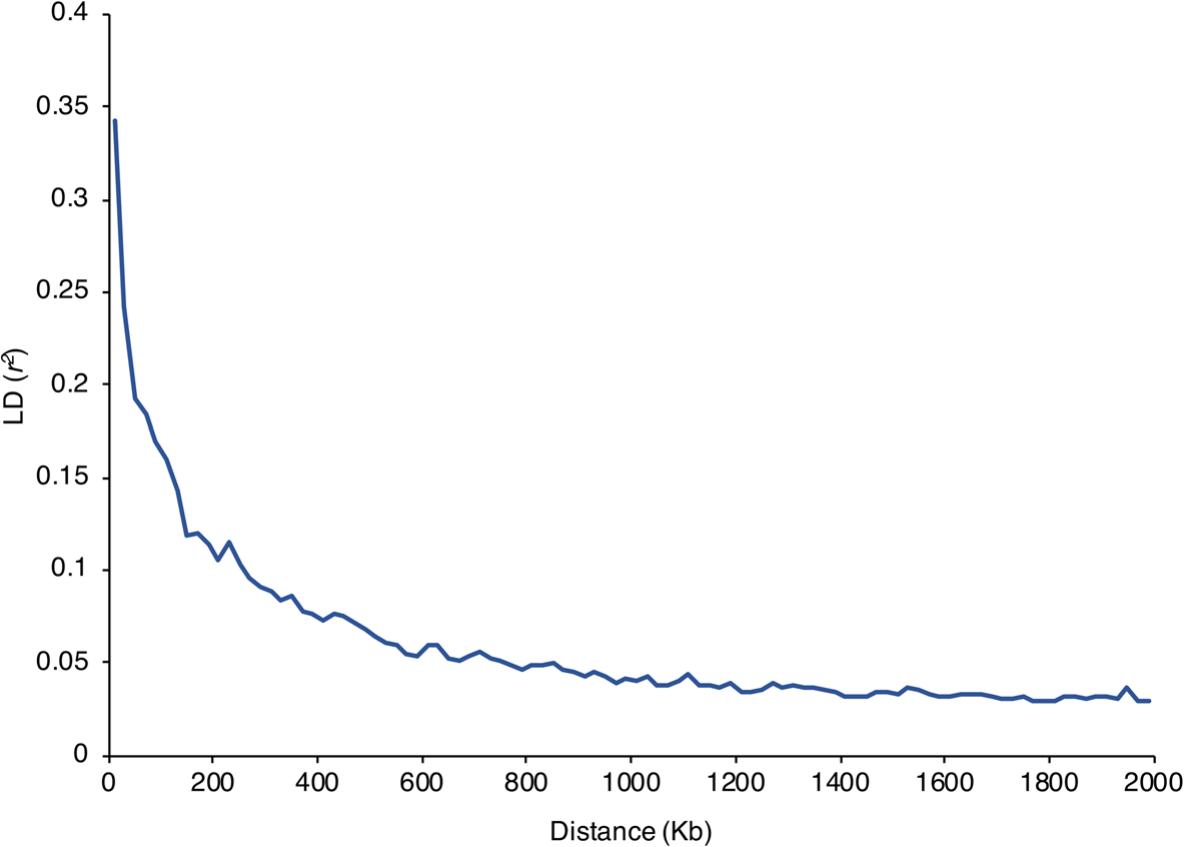
LD pattern and LD decay in the whole panel. The whole genome *r*^2^ values from PLINK were first sorted by *r*^2^ values, and then divided into 100 blocks of 20 kb. The *r*^2^ values in each block were averaged and plotted against the physical distance

For LD decay analysis of the panel, the binned *r*^2^ values were mapped against the physical distance and the distance at which the average of *r*^2^ dropped to half of the maximum value was described as LD decay. The average LD for SNPs at 20 kb distance from each other was 0.34 (*r*^2^), which decayed to its half value (~ 0.17) at around 80 kb (Fig. 4). Additionally, PLINK was also used to calculate chromosome-wise LD between SNPs pairs. At 5 kb from each other, the greatest *r*^2^ was found on chromosome 3 (*r*^2^ = 0.57) and the lowest *r*^2^ was found on chromosome 11 (*r*^*2*^ = 0.27).

### Genome-wide association mapping and candidate loci associated with salt tolerance at flowering stage

**Fig. 5:**
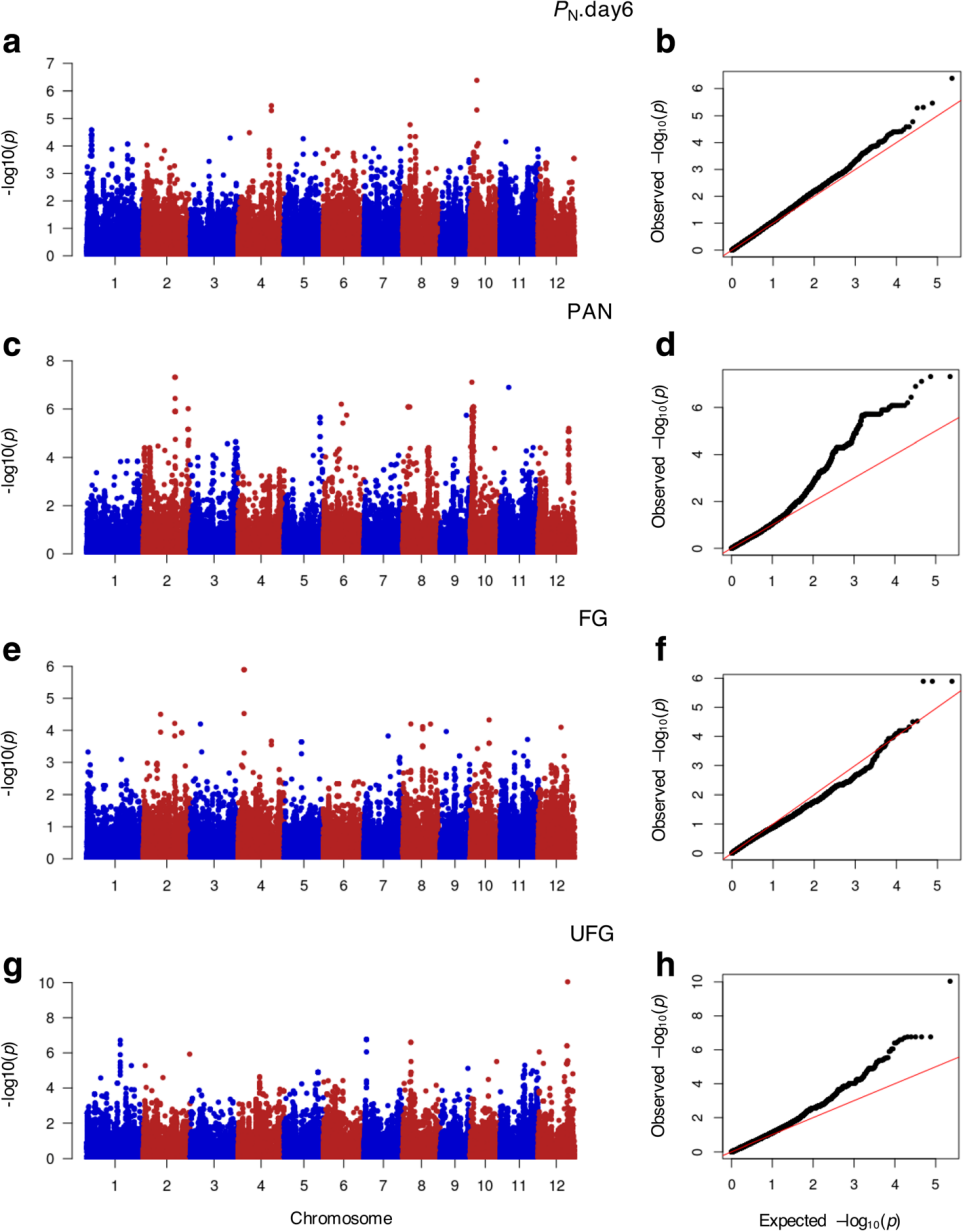
Manhattan and Quantile-quantile (Q-Q) plots of GWAS. GWAS analysis was carried out using SNP markers from the exon region associated with (**a** and **b**) *P*_N_ on day 6 after the salt stress treatment: *P*_N_.day6 (**c** and **d**) PAN (**e** and **f**) FG (**g** and **h**) UFG of 104 accessions as phenotypic data. For Manhattan plots, *x*-axis represents SNP positions across the entire rice genome by chromosome and the *y*-axis is the negative logarithm *p-*value: -log_10_ (*p*) of each SNP. For Q-Q plots, *x*-axis represents expected -log_10_ (*p*) and *y*-axis is observed -log_10_ (*p*) of each SNPs. *P*_N_: net photosynthetic rate; *g*_s_: stomatal conductance; *E*: transpiration rate; *C*_i_: intercellular CO_2_ concentration; CMS: cell membrane stability, TIL: number of tillers per plant; PAN: number of panicles per plant; FG: number of filled grains per plant and UFG: number of unfilled grains per plant

**Fig. 6:**
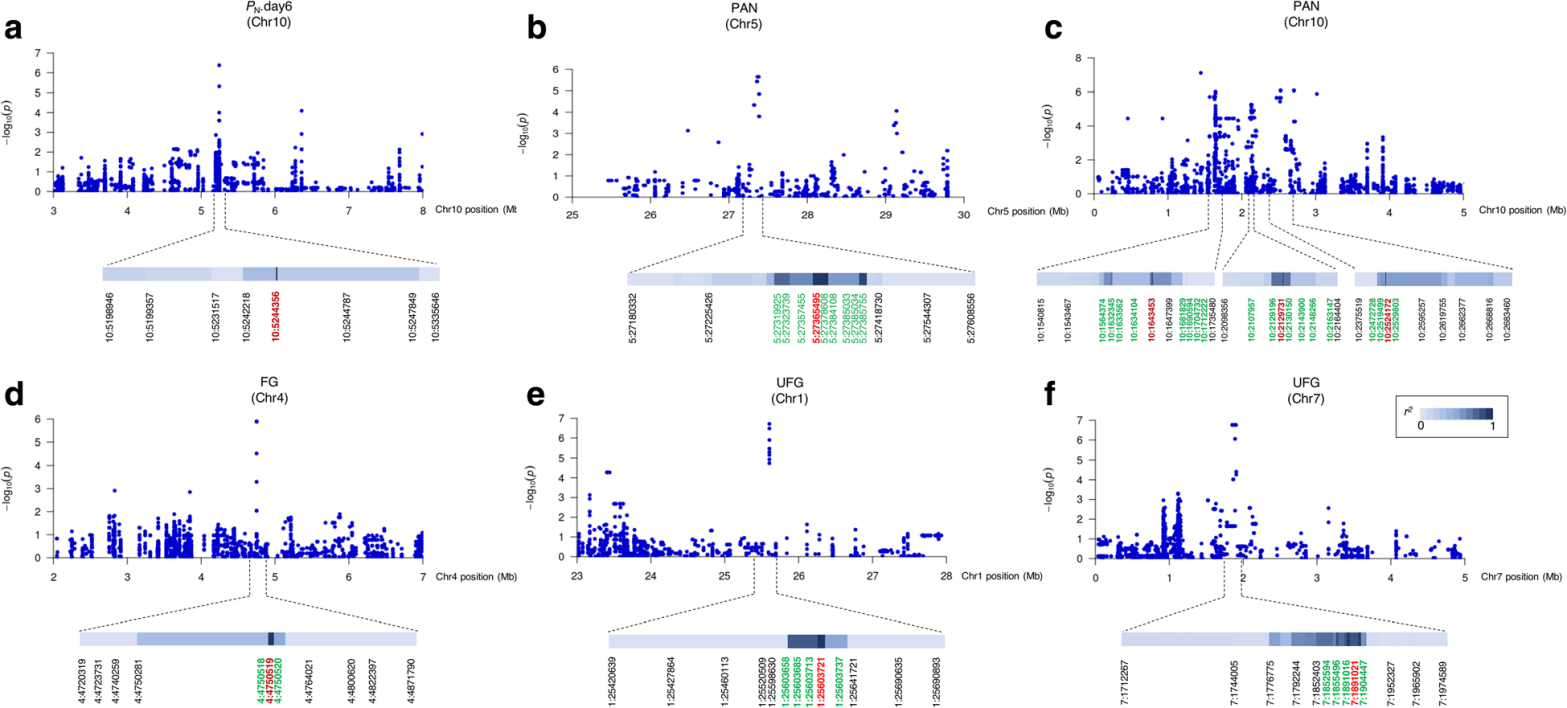
The peak regions on rice chromosomes containing significant SNPs from GWAS of *P*_N_.day6 (**a**), PAN (**b** and **c**), FG (**d**) and UFG (**e** and **f**). The pair-wise LD for the SNP of the lowest *p*-value (red letters) is indicated as *r*^2^ values, where the markers were divided into bins of 5 kb and the *r*^2^ values were averaged and shown as blue bars; the darkest blue represents a value of 1 and the lightest represents a value of 0. The dotted lines denote the regions containing LD blocks that the significant SNPs reside. Examples of other significant SNPs are shown in green letters. Note that the diagram of *r*^2^ values represents all neighboring SNPs present in that region, while it is not proportional to the physical distance of the chromosome. *P*_N_: net photosynthetic rate; PAN: number of panicles per plant; FG: number of filled grains per plant and UFG: number of unfilled grains per plant

To identify potential genes associated with salt stress in Thai rice population, GWAS was performed using SNP data and the phenotypic data of 10 parameters by GEMMA software [51]. Manhattan plots were generated to illustrate the significance of exome-sequencing SNPs associated with each trait. Using the mixed linear model (MLM), after correction for multiple testing, markers with a *q*-value (an FDR adjusted *p*-value) < 0.05 were considered as truly significant. Given that an FDR adjusted *p*-value threshold of 0.05 means that 5% of significant tests would result in false positives and the number of spurious associations was greatly reduced. Altogether, 448 significant SNPs were found from GWA mapping of four traits, and the list of SNP positions, alternate SNPs and candidate loci were presented in Additional file 6: Table S4. A significant SNP was found in the GWA mapping of net photosynthesis at day 6 (*P*_N_.day6), which was located on chromosome 10 (Fig. 5a). There were two SNP peaks on the GWA mapping of PAN on chromosomes 2 and 10 (Fig. 5c). A SNP peak was also found in GWA mapping of FG, which was located on chromosome 4 (Fig. 5e) and there were four SNP peaks in the GWA mapping of UFG, which was on chromosomes 1, 7, 8 and 12 (Fig. 5g). The Q-Q plot of expected and observed *p*-values was delineated and SNPs that had *p*-values deviated from the linear indicated reasonable positives (Fig. 5b, d, f, h). Table 2 listed the loci identified by GWAS that contained multiple neighboring significant SNPs appeared and/or significant SNPs of low *p*-value. Figure 6 and Additional file 7: Figure S3a and b show the regions containing those significant SNPs with the shade color in the blue bar representing the pair-wise LD indicated by *r*^2^ value for the SNP of the lowest *p*-value in that region.

**Table 2:** List of genes identified by GWAS that contained multiple neighboring significant SNPs and/or significant SNPs of low *p*-value

Trait	Chr	SNP	Loc Number	Description
*P*_N._day6	10	1	LOC_Os10g09700	OsWAK110 receptor-like kinase
PAN	2	3	LOC_Os02g40410	expressed protein
		4	LOC_Os02g40420	expressed protein
		1	LOC_Os02g55910	monogalactosyldiacylglycerol synthase
		4	LOC_Os02g56020	methyltransferase
		1	LOC_Os02g56130	PCNA - Putative DNA replicative polymerase clamp
		1	LOC_Os02g56630	OsWAK24 - OsWAK receptor-like protein
	5	1	LOC_Os05g47670	zinc finger, C3HC4
		2	LOC_Os05g47690	reticulon domain containing protein
		1	LOC_Os05g47770	serine/threonine-protein kinase At1g18390 precursor
		1	LOC_Os05g47780	E3 ubiquitin ligase
		4	LOC_Os05g47790	expressed protein
	10	1	LOC_Os10g03620	OsFBX344 - F-box domain containing protein
		36	LOC_Os10g03660	OsFBX345 - F-box domain containing protein
		20	LOC_Os10g03669	expressed protein
		2	LOC_Os10g03730	OsFBX347 - F-box domain containing protein
		2	LOC_Os10g03740	OsFBX348 - F-box domain containing protein
		3	LOC_Os10g03770	expressed protein
		3	LOC_Os10g03780	OsFBX351 - F-box domain containing protein
		1	LOC_Os10g04470	conserved hypothetical protein
		2	LOC_Os10g04480	expressed protein
		25	LOC_Os10g04490	expressed protein
		3	LOC_Os10g04510	expressed protein
		2	LOC_Os10g04520	expressed protein
		1	LOC_Os10g04560	hypothetical protein
		3	LOC_Os10g05050	expressed protein
		6	LOC_Os10g05160	expressed protein
		2	LOC_Os10g05170	OsWAK100 receptor-like kinase
		1	LOC_Os10g05180	26S proteasome regulatory subunit S5A
FG	4	3	LOC_Os04g08749	expressed protein
UFG	1	8	LOC_Os01g45120	expressed protein
	7	2	LOC_Os07g04220	wound and phytochrome signaling involved receptor like kinase
		2	LOC_Os07g04270	hypothetical protein
		1	LOC_Os07g04290	alcohol oxidase-related protein
		1	LOC_Os07g04310	expressed protein
	8	1	LOC_Os08g10330	SHR5-receptor-like kinase
		1	LOC_Os08g10430	NBS-LRR disease resistance protein
		1	LOC_Os08g10440	NBS-LRR disease resistance protein
	12	1	LOC_Os12g36630	universal stress protein domain containing protein

### Comparison of the GWAS prediction and previously reported QTLs

**Fig. 7:**
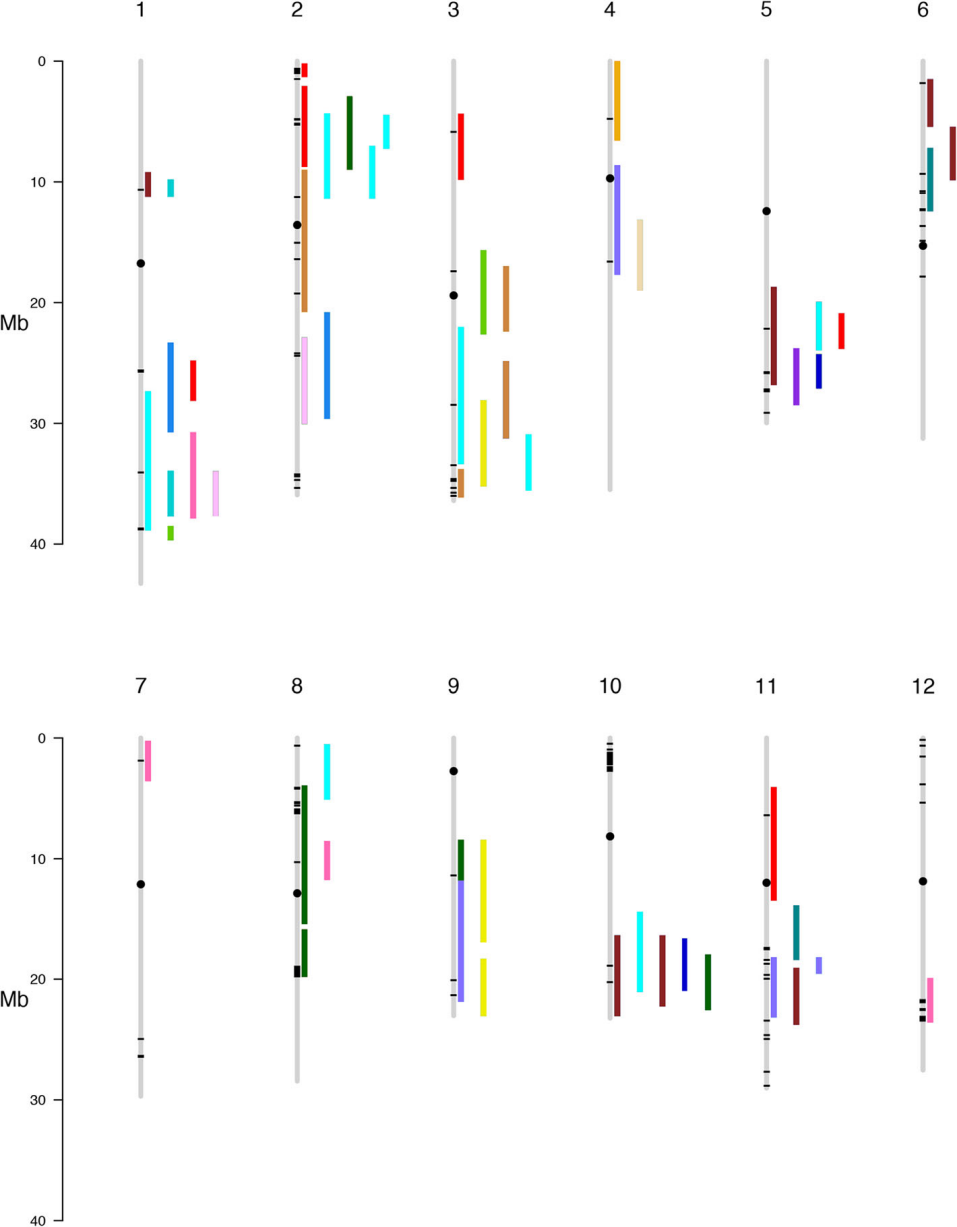
Locations of reported salt QTLs of 12 rice chromosomes that overlap with candidate genes from GWA mapping. The positions of the QTL regions correspond to Table 3. The black lines in each chromosome represent location of significant loci. The boxes on right hand side of each chromosome are salt QTLs identified from previous studies:  Ammar et al. [106],  Bimpong et al. [73],  Ghomi et al. [56],  Hossain et al. [107],  Koyama et al. [19],  Lee et al. [76],  Liang et al. [108],  Lin et al. [75],  Yao et al. [77],  Mohammadi et al. [58],  Prasad et al. [109],  Qiu et al. [74],  Sabouri and Sabouri [80],  Takehisa et al. [78],  UI Haq et al. [110],  Wang et al. [57],  Wang et al. [79]. R 'chromPlot' was used to draw this illustration [111].

Overall, GWAS mapping identified 448 significant SNPs in the exome, which were located on 200 genes (Additional file 6: Table S4). Among these, there were 146 genes co-localized with salinity-related QTLs, which accounted for 73% of all candidate genes covering all rice chromosomes. Figure 7 represents salt-related QTL on which candidate genes were co-located. The top four regions that contained a high number of significant SNPs were found on chromosomes 8, 12, 1 and 2, respectively (Table 3). The region containing the highest density of significant SNPs (100 SNPs) was located between markers RM7027 and RM826 on chromosome 8, which was related with the salt evaluation score (SES) of rice [56]. The second highest density of the significant SNPs (33 SNPs) was located in *qGY12.1* (RM519-RM1103) on chromosome 12, which associated with grain yield (GY) of rice under salt stress at reproductive stage. On chromosome 3, 28 significant SNPs were located in *qGP3* (RM49–RM6712), which involved germination percentage (GP) of rice under salt stress [57]. This region was overlapped with 2 QTLs, including *qPL3.1 s* (RM520–RM570) and *qSHL-3* (RM7000–RM7389). These QTLs were correlated with the panicle length (PL) and shoot length (SHL) [56, 58]. Finally, for salt-treated QTLs of rice involving day of seedling survival (DSS) on chromosome 2, we found that *qDSS2.1* (RM109-RM110) containing 19 significant SNPs identified in this study.

**Table 3:** Summary of reported QTLs and candidate genes from GWA mapping associated with salt tolerance that are co-located to these segments

Chr	QTL (Marker)	QTL Trait	Fragment (Mb)	Co-QTL	Co-QTL Trait	Fragment (Mb)	SNP No.	Loc Number
1	*qSES1, qPH1, qPN1, qPS1, qGPP1* [73] (RM582-RM8094)	SES, PH, PAN, UFG, GPP	2.1	*qSKC-1* [75] (C1211-S2139)	SKC	1.4	1	LOC_Os01g18850
1	*qSTR-1* [77] (RM9-RM128)	STR	7.4	*qSKC1.1* [74] (RM488-RM473)	SKC	3.3	10	LOC_Os01g45120, LOC_Os01g45520, LOC_Os01g59020
				*qPH1.1, qPL1.2, qPF1.5* [107] (RM128-RM472)	PH, PL, PF	7.2		
				*qSDS-1, 99SL* [75, 78] (C813-C86)	DSS, RNC, SHL	3.8		
1	*qPH1.1 s, qPL1.1 s* [58] (RM246-RM431)	PH, PL	11.6	*qST1* [76] (RG109-RZ14)	SES	1.2	3	LOC_Os01g66740, LOC_Os01g66760, LOC_Os01g66800
2	*qDSS2.1* [74] (RM109-RM110)	DSS	1.1	–			19	LOC_Os02g02120, LOC_Os02g02390, LOC_Os02g02410, LOC_Os02g02424, LOC_Os02g02540, LOC_Os02g02640, LOC_Os02g02690, LOC_Os02g02750, LOC_Os02g02930, LOC_Os02g02990
2	*qGY2.1 s* [58] (RM555-RM324)	GY	7.1	*qSST2* [74] (RM211-RM71)	SES	6.7	10	LOC_Os02g09340, LOC_Os02g09420, LOC_Os02g10090, LOC_Os02g10100, LOC_Os02g10200, LOC_Os02g10230, LOC_Os02g19220
				*qSDW-2* [56] (RM279-RM6911)	SDW	6.1		
				*qFRSP2.1 s, qSPFR2.1 s* [58] (RM423-RM174)	FRSP, SPFR	2.7		
				*qPL2.1 s* [58] (RM174-RM424)	PL	4.4		
2	*qPL-2 2* [80] (RM5699-RM262)	PL	11.1	–			3	LOC_Os02g25630, LOC_Os02g27690, LOC_Os02g32480
2	*qNAK-2* [77] (RM262-RM318)	RNAK	8.8	00SW [78] (C560B-C1408)	TIL	7.2	9	LOC_Os02g40120, LOC_Os02g40410, LOC_Os02g40420
3	*qDSS3* [74] (RM489-RM7)	DSS	5.5	–			2	LOC_Os03g11280
3	*qST3* [76] (RZ585-RG179)	SES	7	*qFWRO-3b* [80] (RM6283-RM6832)	RFW	5.4	2	LOC_Os03g30510, LOC_Os03g35340
3	*qPH3.1 s* [58] (RM487-RM130)	PL	11.4	*qNAUP-3* [80] (RM5626-RM416)	NAUP	6.4	1	LOC_Os03g49860
3	*qGP-3* [57] (RM49-RM6712)	GP	7.1	*qPL3.1* s [58] (RM520-RM570)	PL	4.7	28	LOC_Os03g58764, LOC_Os03g61080, LOC_Os03g61110, LOC_Os03g61130, LOC_Os03g61140, LOC_Os03g61150, LOC_Os03g61220, LOC_Os03g62388, LOC_Os03g62400, LOC_Os03g62430, LOC_Os03g63250, LOC_Os03g63680
				*qSHL-3* [80] (RM7000-RM7389)	SHL	2.4		
4	TEL4S-RM261 [19]	SKC	6.6	–			3	LOC_Os04g08749
4	RG190-RG449 [110]	SNC, SKC	9.1	*qDSRs4–2* [108] (RM307-RM471)	DSR	5.9	2	LOC_Os04g28170, LOC_Os04g28190
5	*qDTF5* [73] (RM430-RM274)	HD	8.2	*qDWRO-5b* [80] (RM440-RM480)	RDW	7.4	11	LOC_Os05g37870, LOC_Os05g44290, LOC_Os05g44320, LOC_Os05g44330, LOC_Os05g44370
				*qSST5* [74] (RM161-RM3476)	SES	2.9		
				*qSDM5–5* [109] (RZ70-RZ225)	BM	2.9		
5	*QTL44* [106] (M233B-RM334)	SES	4.7	–			9	LOC_Os05g47670, LOC_Os05g47690, LOC_Os05g47770, LOC_Os05g47780, LOC_Os05g47790
6	*qSES6, qTN, qPN6, qGPP6, qTGW6, qGY6* [73] (RM586-RM253)	SES, TIL, PAN, GY, 1000GW	4.0	–			1	LOC_Os06g04190
6	*qDRW6* [79] (RM5531-RM3183)	RDW	5.3	*qTN6, qGY6* [73] (RM253-RM527)	TIL, GY	4.4	5	LOC_Os06g16400, LOC_Os06g18980, LOC_Os06g19300, LOC_Os06g21210, LOC_Os06g21230
7	*qPH7.1, qTNI7.2* [107] (RM51-RM1243)	PL, TIL	3.35	–			6	LOC_Os07g04220, LOC_Os07g04270, LOC_Os07g04290, LOC_Os07g04310
8	*qSTW8.1 s* [58] (RM407-RM310)	STDW	4.6	–			6	LOC_Os08g02050, LOC_Os08g07400, LOC_Os08g07430
8	*qSNK-8* [56] (RM3572-RM404)	SNK	11.5	*qTN8.1, qBM8.2* [107] (RM3215-RM44)	TIL, BM	3.2	11	LOC_Os08g09200, LOC_Os08g09260, LOC_Os08g09715, LOC_Os08g10330, LOC_Os08g10430, LOC_Os08g10440, LOC_Os08g10570, LOC_Os08g10630, LOC_Os08g16880
8	*qSTR-8* [56] (RM7027-RM8264)	STR	4	–			100	LOC_Os08g30730, LOC_Os08g30740, LOC_Os08g30770, LOC_Os08g30780, LOC_Os08g30790, LOC_Os08g30800, LOC_Os08g30810, LOC_Os08g30820, LOC_Os08g30870, LOC_Os08g30900, LOC_Os08g30910, LOC_Os08g30930, LOC_Os08g31000, LOC_Os08g31040, LOC_Os08g31080, LOC_Os08g31110, LOC_Os08g31120, LOC_Os08g31130, LOC_Os08g31180, LOC_Os08g31320, LOC_Os08g31340, LOC_Os08g31410, LOC_Os08g31420, LOC_Os08g31450, LOC_Os08g31460, LOC_Os08g31470, LOC_Os08g31550, LOC_Os08g31600, LOC_Os08g31620, LOC_Os08g31690, LOC_Os08g31700, LOC_Os08g31740, LOC_Os08g31800, LOC_Os08g31814, LOC_Os08g31840, LOC_Os08g31980
9	*qGP-9* [57] (RM219-RM7048)	SG	8.5	*qRL-*9 [56] (RM219-RM7038)	RL	3.4	1	LOC_Os09g18530
9	RZ206-RG358 [110]	LWC	10.1	*qIR-9* [57] (RM3320-RM2144)	IR	4.7	2	LOC_Os09g34110, LOC_Os09g36976
10	*qPS10, qGPP10* [73] (RM184-RM590)	UFG, GPP	*6.7*	*qDTF10.1 s, qSPFR2.10s, qTGW10.1 s* [58] (RM271-RM484)	DTF, SPFR, 1000GW	6.7	2	LOC_Os10g35436, LOC_Os10g38020
				*qDTF10.1, qTGW10* [73] (RM184-RM228)	HD, 1000GW	5.9		
				*qSDM-10* [109] (RZ625-RZ500)	BM	4.4		
				*qSKC-10b* [56] (RM258-RM333)	SKC	4.4		
11	*qDSS11* [74] (RM167-RM3428)	DSS	9.4	–			1	LOC_Os11g11510
11	*qDRW11* [79] (RM6091-RM229)	RDW	4.5	–			2	LOC_Os11g29970, LOC_Os11g30220
11	RG16-RG1109 [110]	LWC	5	RG16-CDO365 [110]	LWC	1.4	5	LOC_Os11g31480, LOC_Os11g31810, LOC_Os11g33230, LOC_Os11g33942
11	*qTN11, qPN11, qGPP11, qGY11* [73] (RM457-RM254)	TIL, PAN, GPP, GY	4.7	–			2	LOC_Os11g39330, LOC_Os11g40420
12	*qGY12.1* [107] (RM519-RM1103)	GY	3.7	–			33	LOC_Os12g35770, LOC_Os12g35820, LOC_Os12g35850, LOC_Os12g35920, LOC_Os12g36630, LOC_Os12g38051, LOC_Os12g37920, LOC_Os12g36690, LOC_Os12g37720, LOC_Os12g37740 LOC_Os12g37760, LOC_Os12g37770, LOC_Os12g37860, LOC_Os12g37870, LOC_Os12g37890

## Discussion

### Variations of the salt tolerance of rice at the flowering stage

Salt-tolerance of rice is a dynamic trait affected by growing stage and genotype [59, 60]. This study is the first large-scale tolerance evaluation and GWAS focusing on salt stress at the reproductive stage of rice [61]. In this study, we applied salt stress to flowering rice and evaluated five parameters of photosynthetic performance (photosynthetic rate, *P*_N_; stomatal conductance, *g*_s_; transpiration rate, *E*; and intercellular CO_2_ concentration, *C*_i_), cell membrane stability, CMS and four parameters of yield-related traits (number of tillers per plant, TIL; number of panicles per plant, PAN; number of filled grains per plant, FG; and number of unfilled grains per plant, UFG). On average, the stability indices of photosynthesis performance and CMS decreased under salt stress, while increases of *C*_i_ were found, which were similar to the yield parameter, UFG (Table 1 and Fig. 1). For *P*_N_, *E* and *g*_*s*_, these findings agreed with previous studies describing salt-induced photosynthesis reduction in rice seedlings [62–64]. Consistently, during the reproductive stage, Moradi and Ismail [65] found that *P*_N_, *E* and *g*_*s*_ were inhibited in the flag leaf under salinity. Additionally, the salt-sensitive rice cultivar IR29 displayed higher *C*_*i*_ than others when exposed to salt stress during both seedling and reproductive stages. As indicated by Burghardt et al. [27], GWAS would have power to discover genes affecting the trait of interest in large of phenotypic variation greater than small of phenotypic variation. In this study, large variation was observed in *P*_N_ in photosynthesis performance; and PAN, FG and UFG in yield-related traits under salt stress (Fig. 1). Correspondingly we found high detection power of association mapping in these parameters, whereas GWAS for the other parameters that exhibited lower variation was not successful (Fig. 5).

### Rice genome variations and genome-wide association mapping

Using efficient, high precision exome capture and sequencing, we have identified 112,565 SNPs. Previous studies used SNP array to identify SNP markers for GWAS in rice and yielded fewer SNP markers when compared with our study [33, 38, 66]. The present study, however, focused on exonic regions, which are specific sequences in the genome while accounting for only one-sixth of the rice genome, resulted in more than 100,000 SNPs. Although several statistically robust models have been developed for GWAS [67], population structure can limit its effectiveness [68, 69]. Our Thai rice population belongs to the *indica* group (Additional file 5: Figure S2). While its size is relatively small compared human studies, GWAS with similar population size has been effective in *Arabidopsis* [24] and rice [26]. Indeed, diversified composition of our population, lack of strong subpopulation structure, and its homozygosity facilitated GWAS [26, 38].

### Candidate genes associated with salt tolerance of rice at the flowering stage

Altogether, GWAS using this Thai rice population leveraged more than 110,000 SNPs to identify 448 SNPs associated with salt tolerance, which were located in 200 loci in the rice genome. As presented in Table 3, 73% of candidate genes from association mapping associated with salt stress were located within salinity tolerance QTLs identified in bi-parental segregating populations.

Functional annotation of the 200 identified genes revealed a number of plausible candidates. The gene annotations we employed relay on the presence of a protein domain or of a homolog with a known function in rice and other crop species such as maize or sorghum [70, 71], as well as Arabidopsis [72]. Two chromosomes contained the highest number of reported salt QTLs (Table 3 and Fig. 7) overlapping with 7 of our candidate loci: chromosome 1, which included 16 QTLs [58, 73–78, 107] and chromosome 2, which included 10 QTLs [56, 58, 74, 77, 79, 80]. The nature of the candidate genes indicates that different molecular and cellular strategies have evolved to favor survival during salt stress [81]. Several genes belong to the receptor kinase family (LOC_Os01g66740, LOC_Os01g66760, LOC_Os02g02120 and LOC_Os02g56630), encoding signaling factors during environmental stresses [82, 83]. LOC_Os01g18850, one of candidate genes detected by GWA mapping of UFG trait, encodes SQUAMOSA promoter binding protein-like (SPL) transcription factor (TF), a plant specific TF, whose function was suggested to affect a broad range growth and development processes, including flower development [84] and 19 *SPL* genes were identified in rice [85]. The role of *SPL* gene in salt stress response has been studied by Mao et al. [86]. The 31 *SPL* genes were identified in maize and the expression profiles of *SPLs* revealed that most *SPL* genes were induced under salt stress condition.

A candidate gene identified here encodes cytochrome P450 monooxygenases (LOC_Os01g59020). This enzyme, common to bacteria, plants and humans, shares a common catalytic center, a heme with an iron coordinated to the thiolate of a conserved cysteine [87]. They oxidize disparate substrates through activation of molecular oxygen. The plant P450 gene superfamily plays crucial roles in plant metabolic processes [88]. Narusaka et al. [89] analyzed the expression of 49 Arabidopsis P450 genes under various stresses, including salt stress, and found that 29 P450-genes were induced by various stresses. In the CYP709B subfamily of P450, a *cyp709b3* Arabidopsis mutant showed sensitivity to salt stress during germination and high salt-damage at the seedling stage [90]. In rice, Tamiru et al. [91] reported that a P450 gene, *OsDSS1* located on chromosome 3 was involved in growth and drought stress responses. Compared to WT, the *dss1* rice mutant exhibited improved recovery after germination under drought stress. Additionally, ectopic expression of the P450 gene *PtCYP714A3* from *Populus trichocarpa* was studied in rice. Transgenic rice expressing *PtCYP714A3* was semi-dwarf with improved tolerance to salt and osmotic stress, resulting in higher survival rates than WT [92].

Interestingly, several novel candidate loci with 144 significant SNPs identified from this GWA mapping were found on chromosome 10, in which no salt QTL was reported. This represents the highest density of significant SNPs found in the same LD block (Fig. 6c). Interestingly, seven of these SNP-associated genes encoded F-box domain containing proteins (LOC_Os10g03620, LOC_Os10g03660, LOC_Os10g03730, LOC_Os10g03740, LOC_Os10g03780, LOC_Os10g03930 and LOC_Os10g05500). Previous studies have reported the role of F-box proteins in regulating various abiotic stress responses in Arabidopsis, wheat and rice [93–97]. A conserved N-terminal F-box domain (40–50 amino acids), is a component of the multi-subunit of ubiquitin E3 ligase, an enzyme in the last step of the ubiquitination pathway [98, 99]. The rice genome harbors more than 600 F-box genes whose divergence is consistent with adaptive roles [100] and regulation of 25 of these genes responds to salinity stress [94]. Rice seedlings overexpressing F-box protein gene, *MAIF1* reduced inhibition of root growth and tolerance under salt stress compared with WT [97]. Salt induced the expression of *OsMsr9*, a novel rice putative F-box containing protein, especially in the panicle. Overexpression of *OsMsr9* increased root length, shoot length and survival rate under salt stress [101].

Moreover, SNP with the lowest *p*-value (9.04 × 10^− 11^) found on chromosome 12 of GWA mapping of UFG (Table 2, Additional file 7: Figure S3d) was located in LOC_Os12g36630, which was annotated as a universal stress protein (USP) domain containing protein. In fact, USP genes are widely distributed across many organisms including plant, which encode a protein containing the 140–160 highly conserved residues of the Universal Stress Protein A domain (USPA, Pfam accession number PF00582). These genes were reported as environmental stress-responsive genes and played role in the ability of plant to respond to the stresses [102, 103]. To date, there are no report on the role of USP genes in salt-treated rice. However, in the study on *OsUsp1* in rice under oxygen deficiency condition, it was found that *OsUsp1* expression was strongly induced within 1 h of submergence and it played a role in ethylene-mediated stress adaptation in rice [104]. Furthermore, the role of the USP protein (At3g53990: *AtUsp*) in enhancing oxidative stress has been reported in the plant model Arabidopsis [105]. They found that the over-expression of *AtUSP* conferred a strong tolerance to oxidative stress, primarily via its chaperone function.

## Conclusion

High quality genotyping data from high-throughput sequencing combined with robust statistical analysis, enables GWA mapping of complex quantitative traits. We conducted GWAS for salt tolerance during rice reproduction based on high-density SNPs in exon regions using *indica* Thai accessions. Altogether, the significant SNPs were located on 200 loci distributed among all rice chromosomes. Our GWA mapping was highly consistent with previous salt tolerance QTL mapping studies conducted in bi-parental populations. Overall, more than 73% of the candidate genes controlling salt tolerance identified in our GWAS overlap with the salt QTLs. While many are novel, their annotation is consistent with potential involvement in plant salt tolerance and in related agronomic traits. These significant SNPs greatly help narrow down the region within these QTLs where the likely underlying candidate genes can be identified. Knowledge on the varieties with high salt tolerance, as well as the associated SNPs from this study, will be useful for future improvement of rice yield productivity under salt stress.

## Additional files

Additional file 1: **Table S1.** List of all rice accessions used for SNP genotyping. Rice accessions are tabled with their geographical locations, GS No., parental lines/parental relations and heterozygosity. (XLSX 19 kb)

Additional file 2: **Table S2.** List of rice accessions and phenotypic data used for association mapping. The average values of four biological replicates are shown. (XLSX 50 kb)

Additional file 3: **Figure S1.** Frequency distribution of *P*_N_: net photosynthetic rate; *g*_s_: stomatal conductance; *E*: transpiration rate; *C*_i_: intercellular CO_2_ concentration, CMS: cell membrane stability, TIL: number of tillers per plant; PAN: number of panicles per plant; FG: number of filled grains per plant and UFG: number of unfilled grains per plant for 104 rice accessions under control (a and b) and salt stress conditions (c and d). (PDF 306 kb)

Additional file 4: **Table S3.** Pearson’s correlation coefficients of the phenotypic traits measured in 104 rice accessions. Data are from salt susceptible indices of each trait. Shaded values are significant at *p* < 0.05, *p* < 0.01, or *p* < 0.001, darker shading indicates higher significance. (XLSX 11 kb)

Additional file 5: **Figure S2.** Population structure of 190 rice association panels, which consisted mostly of the *indica* accessions. (TIF 5827 kb)

Additional file 6: **Table S4.** List of significant SNPs from GWA mapping for various traits under control and stress conditions using compressed MLM. Details are also given for MSU Locus ID and their putative functions from where the respective SNP was selected. (XLSX 39 kb)

Additional file 7: **Figure S3.** The peak regions on rice chromosomes containing significant SNPs from GWAS of PAN (a and b) and UFG (c and d). The pair-wise LD for the SNP of the lowest p-value (red letters) is indicated as r2 values, where the markers were divided into binds of 5 kb and the r2 values were averaged and shown as blue bars; the darkest blue represents a value of 1 and the lightest blue represents a value of 0. The dotted lines denote the regions containing LD blocks that the significant SNPs reside. Examples of other significant SNPs are shown in green letters. Note that the diagram of r2 values represents all neighboring SNPs present in that region, while it is not proportional to the physical distance of the chromosome. (TIFF 9604 kb)

## Abbreviations

1000GW: 1000-grain weight
BM: Biomass
*C*_i_: Intercellular CO_2_ concentration
CMS: Cell membrane stability
DAT: Day after treatment
DSR: Dead seedling rate
DSS: Days of seedling survival
DTF: Day to flower
*E*: Transpiration rate
EC: Electrical conductivity
FG: Number of filled grains per plant
FRSP: Number of fertile spikelets
GP: Germination Percentage
GPP: Grains per panicle
*g*_s_: Stomatal conductance
GWAS: Genome-wide association study
GY: Grain yield
HD: Heading date
IR: Imbibition rate
LD: Linkage Disequilibrium
LWC: Leaf water content
MAF: Minor allele frequency
MLM: Mixed linear model
NAUP: Na^+^ uptake
PAN: Number of panicles per plant
PF: Pollen fertility
PH: Plant height
PL: Plant length
*P*_N_: Net photosynthetic rate
Q-Q: The quantile–quantile
QTLs: Quantitative trait loci
RDW: Root dry weight
RFW: Root fresh weight
RL: Root length
RNAK: Root Na^+^/K^+^ ratio
RNC: Root Na^+^ concentration
SDW: Shoot dry weight
SES: Salt evaluation score
SHL: Shoot length
SKC: Shoot K^+^ concentration
SNC: Shoot Na^+^ concentration
SNK: Shoot Na^+^/K^+^ ratio
SNP: Single nucleotide polymorphism
SPFR: Spikelet fertility
STDW: Straw dry weight
STR: Standard tolerance ranking
TIL: Number of tillers per plant
UFG: Number of unfilled grains per plant
