# Supplementary material for: Genome-wide association study for salinity tolerance at the flowering stage in a panel of rice accessions from Thailand
Source: BMC Genomics. 2019 Jan 22;20:76. doi: 10.1186/s12864-018-5317-2 (PMC6343365; doi:10.1186/s12864-018-5317-2)
Supplement: Supplementary file 3 — Figure S1. Frequency distribution of PN: net photosynthetic rate; gs: stomatal conductance; E: transpiration rate; Ci: intercellular CO2 concentration, CMS: cell membrane stability, TIL: number of tillers per plant; PAN: number of panicles per plant; FG: number of filled grains per plant and UFG: number of unfilled grains per plant for 104 rice accessions under control (a and b) and salt stress conditions (c and d). (PDF 306 kb) [file 12864_2018_5317_MOESM3_ESM.pdf]

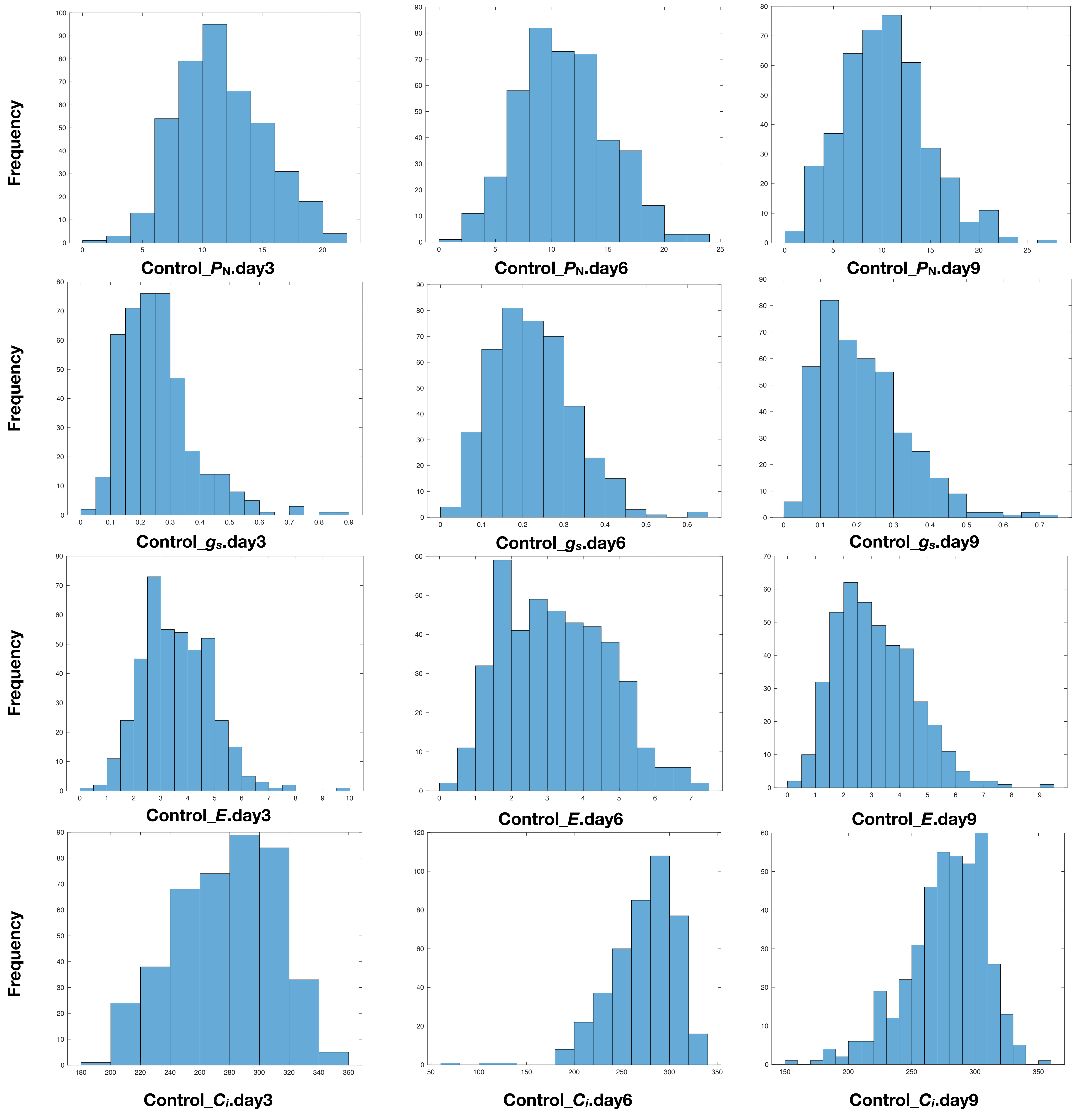

Figure S1a P_N: Net photosynthetic rate; g_s: Stomatal conductance; E: Transpiration rate; C_i: Intercellular CO_2 concentration for 104 rice accessions under control condition

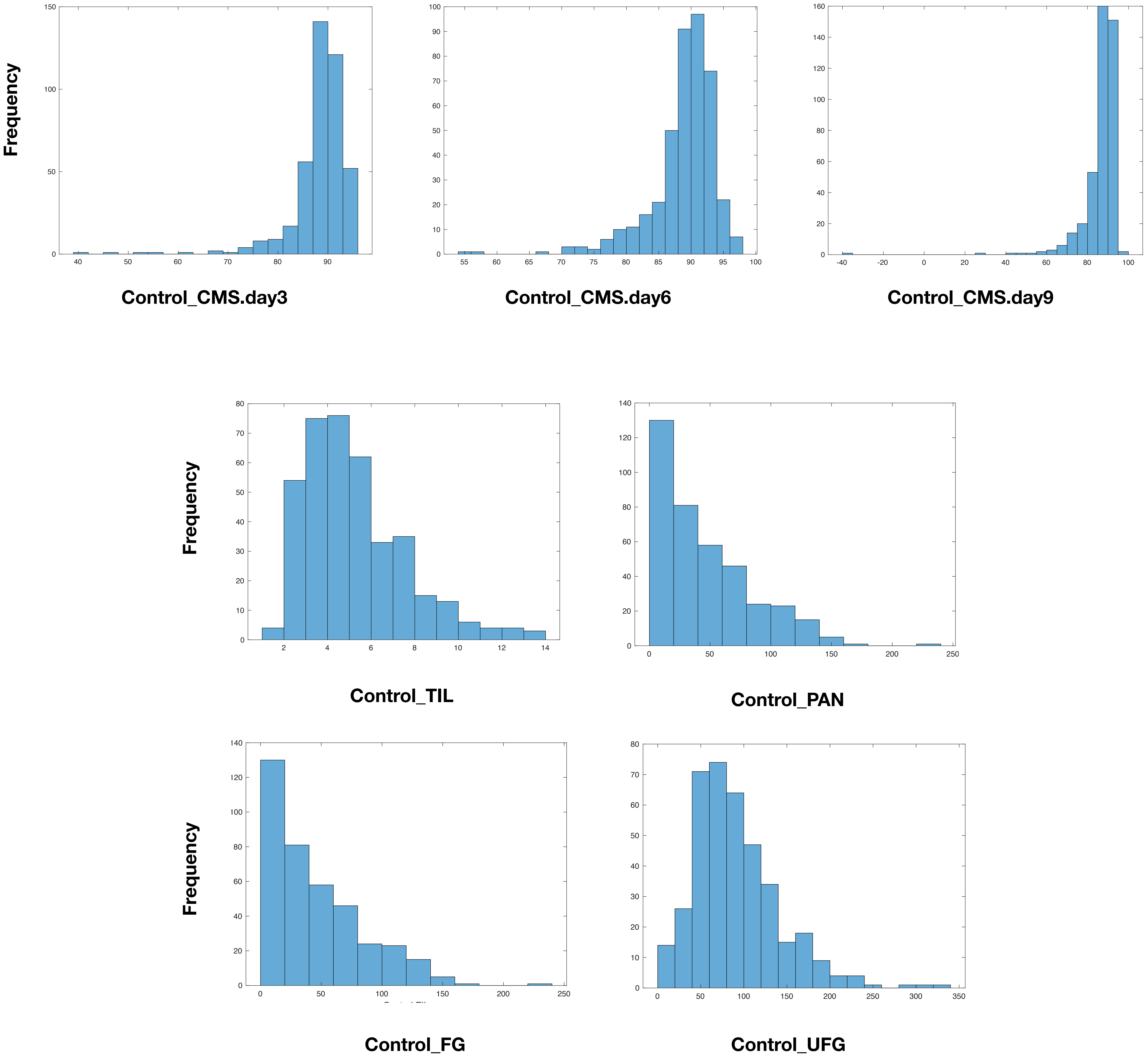

Figure S1b CMS: cell membrane stability; TIL: Number of tillers per plant; PAN: Number of panicles per plant; FG: Number of filled grains per plant and UFG: number of unfilled grains per plant for 104 rice accessions under control condition

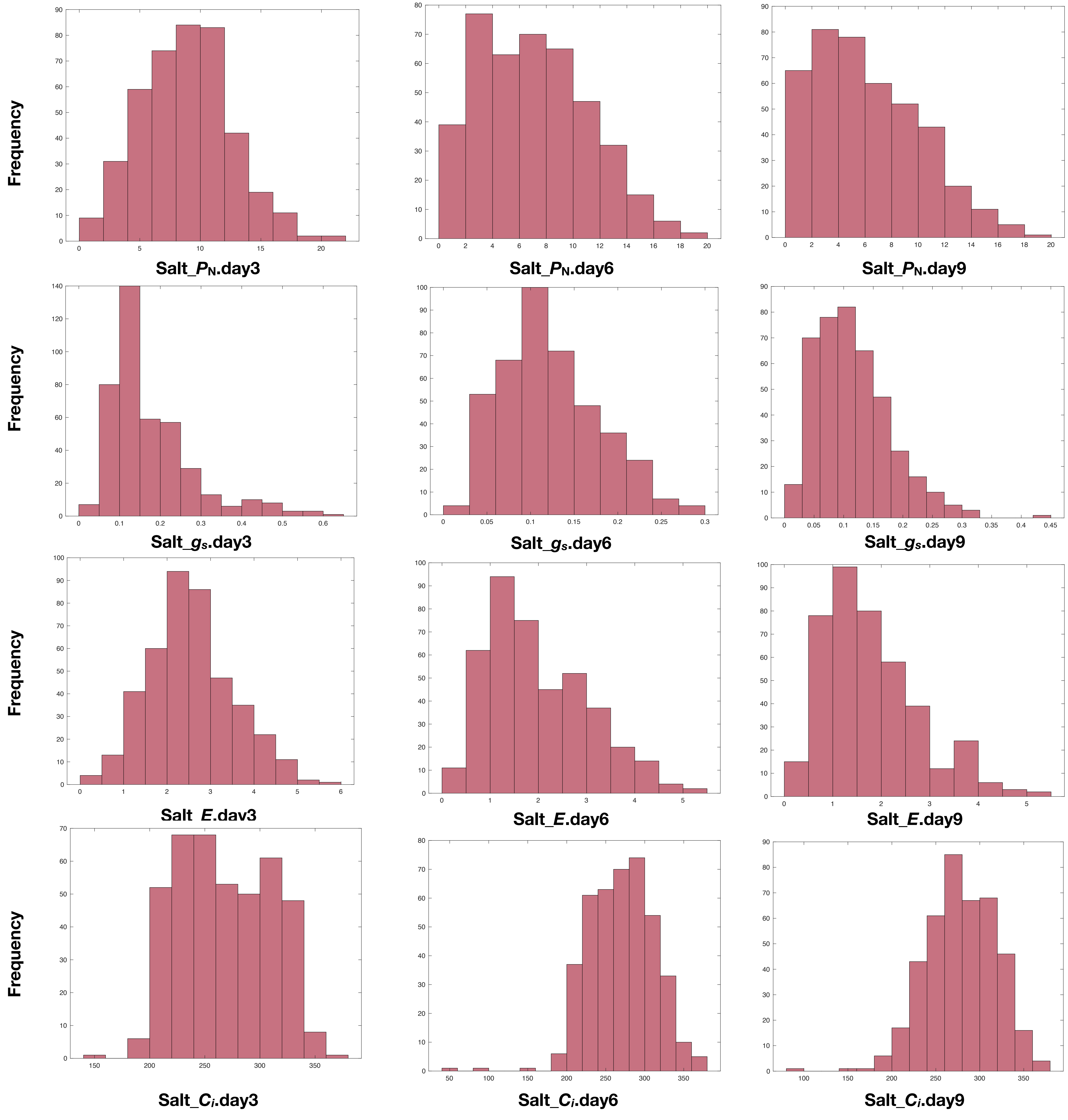

Figure S1c P_N: Net photosynthetic rate; g_s: Stomatal conductance; E: Transpiration rate; C_i: Intercellular CO_2 concentration for 104 rice accessions under salt stress condition

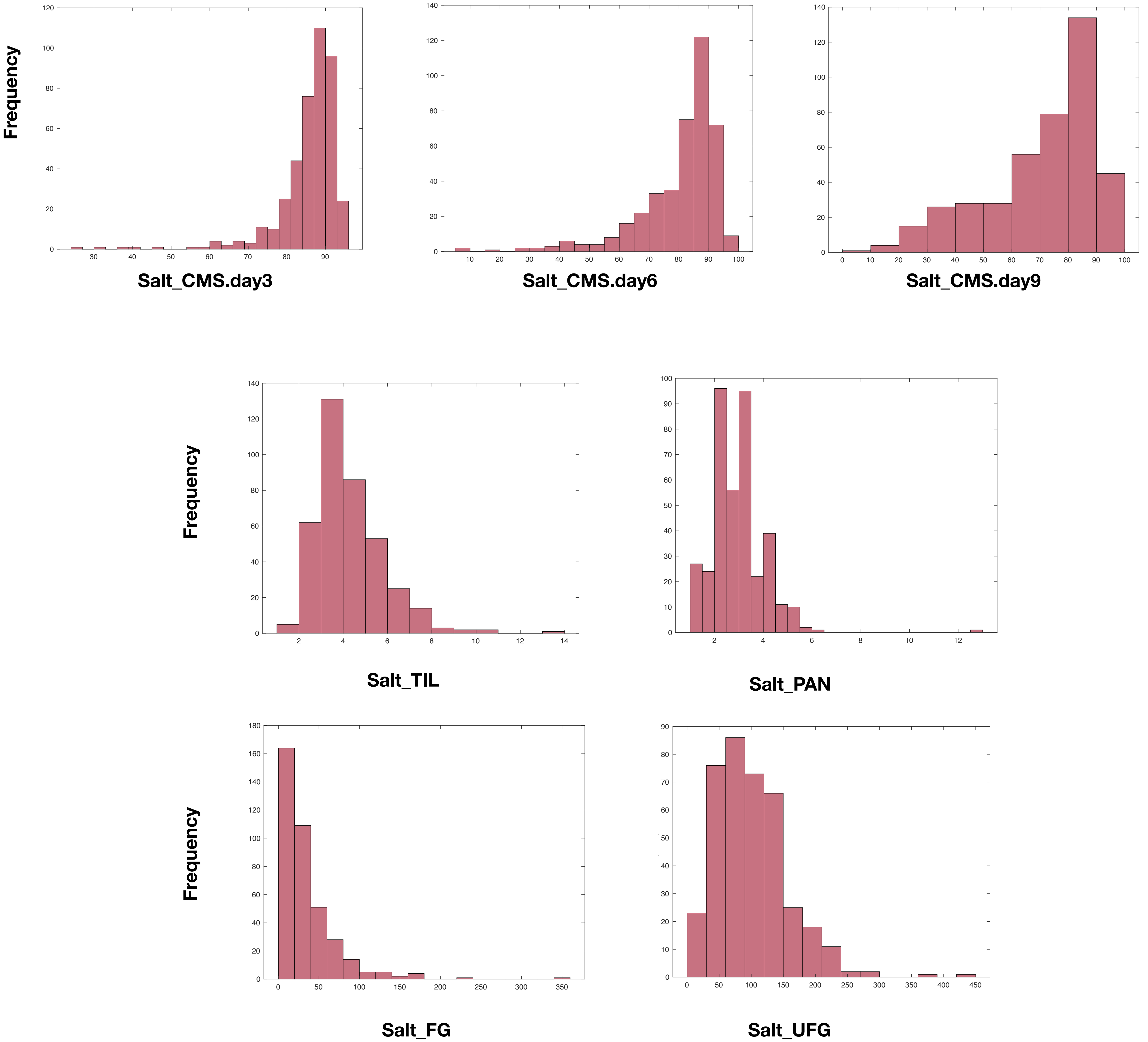

Figure S1d CMS: cell membrane stability; TIL: Number of tillers per plant; PAN: Number of panicles per plant; FG: Number of filled grains per plant and UFG: number of unfilled grains per plant for 104 rice accessions under salt stress condition
