# Supplementary figures and images for: Genome-wide association study for salinity tolerance at the flowering stage in a panel of rice accessions from Thailand
Source: BMC Genomics. 2019 Jan 22;20:76. doi: 10.1186/s12864-018-5317-2 (PMC6343365; doi:10.1186/s12864-018-5317-2)

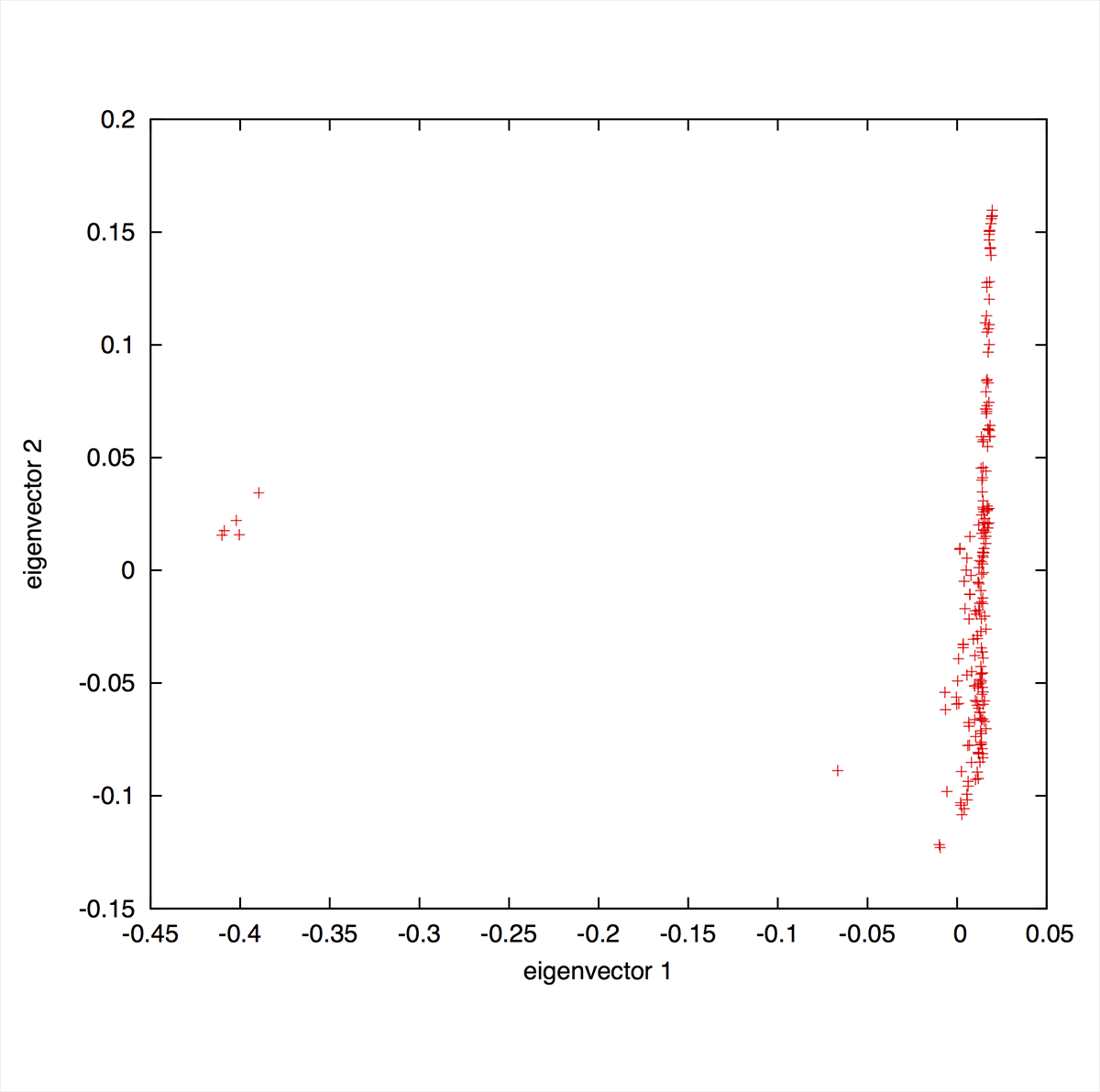

Supplement: Supplementary file 5 — Figure S2. Population structure of 190 rice association panels, which consisted mostly of the indica accessions. (TIF 5827 kb) [file 12864_2018_5317_MOESM5_ESM.tif]
